# Identification of Subnanometric Ag Species, Their Interaction with Supports and Role in Catalytic CO Oxidation

**DOI:** 10.3390/molecules21040532

**Published:** 2016-04-22

**Authors:** Yulia Kotolevich, Ekaterina Kolobova, Evgeniy Khramov, Jesús Efren Cabrera Ortega, Mario H. Farías, Yan Zubavichus, Rodolfo Zanella, Josué D. Mota-Morales, Alexey Pestryakov, Nina Bogdanchikova, Vicente Cortés Corberán

**Affiliations:** 1 Departamento de Fisicoquímica de Nanomateriales, Centro de Nanociencias y Nanotecnología, Universidad Nacional Autónoma de México (UNAM), Ensenada 22860, Mexico; Julia.Kotolevich@gmail.com (Y.K.); mario@cnyn.unam.mx (M.H.F.); jmota@cnyn.unam.mx (J.D.M.-M.); nina@cnyn.unam.mx (N.B.); 2 Department of Physical and Analytical Chemistry, Tomsk Polytechnic University, Tomsk 634050, Russia; ekaterina_kolobova@mail.ru (E.K.); pestryakov2005@yandex.ru (A.P.); 3 National Research Center “Kurchatov Institute”, Moscow 123182, Russia; evxramov@gmail.com (E.K.); yzubav@googlemail.com (Y.Z.); 4 Departamento de Fisica Aplicada, Centro de Investigación Científica y de Educación Superior de Ensenada, Ensenada 22860, Mexico; efren507.507@gmail.com; 5 Centro de Ciencias Aplicadas y Desarrollo Tecnológico, Universidad Nacional Autónoma de México (UNAM), México, DF 04510, Mexico; rodolfo.zanella@ccadet.unam.mx; 6 CONACYT Research Fellow at Centro de Nanociencias y Nanotecnología, UNAM, Ensenada 22860, Mexico; 7 Institute of Catalysis and Petroleumchemistry (ICP), Spanish Council for Scientific Research (CSIC), Madrid 28049, Spain

**Keywords:** silver catalysts, subnanometer species, CO oxidation, sensitivity to pretreatments, interaction with support

## Abstract

The nature and size of the real active species of nanoparticulated metal supported catalysts is still an unresolved question. The technique of choice to measure particle sizes at the nanoscale, HRTEM, has a practical limit of 1 nm. This work is aimed to identify the catalytic role of subnanometer species and methods to detect and characterize them. In this frame, we investigated the sensitivity to redox pretreatments of Ag/Fe/TiO_2_, Ag/Mg/TiO_2_ and Ag/Ce/TiO_2_ catalysts in CO oxidation. The joint application of HRTEM, SR-XRD, DRS, XPS, EXAFS and XANES methods indicated that most of the silver in all samples is in the form of Ag species with size <1 nm. The differences in catalytic properties and sensitivity to pretreatments, observed for the studied Ag catalysts, could not be explained taking into account only the Ag particles whose size distribution is measured by HRTEM, but may be explained by the presence of the subnanometer Ag species, undetectable by HRTEM, and their interaction with supports. This result highlights their role as active species and the need to take them into account to understand integrally the catalysis by supported nanometals.

## 1. Introduction

The discovery of the extraordinary catalytic activity of gold nanoparticles for oxidation reactions compared to that of the bulk metal sparked the interest on the influence of the metal particle size at the nanometer scale on the catalytic performance of noble metal-based catalysts. In the literature, there are publications describing catalysts active at low temperature that contain both particles undetectable by ТЕМ and, in the other extreme, very large particles [1,2,3,4,5,6,7,8]. Difficulties in the interpretation of such results are associated with the limited information provided by the routine physical and chemical research methods of characterization (e.g., XRD and TEM).

Over the last 20 years, only a few papers have explained the atypical behavior of catalysts by the presence of ultra-small particles of gold, *i.e.*, subnanometer clusters [9,10,11,12,13] and atomically dispersed single-site cations [14,15]. Modern microscopes, such as aberration-corrected high angle annular dark field scanning transmission microscope (ac-HAADF/STEM), enable one to capture ultra-small particles and atoms [14,16]. Another approach for subnanometer clusters’ detection is by combining the use of multiple research methods, both more common ones (TPR, UV-VIS) and advanced ones (EXAFS, XANES, XPS, DFT).

Flytzani-Stephanopoulos *et al.* leached out Au NPs and showed that gold nanoparticles on various supports are not active in the water-gas shift (WGS) reaction [14,17,18,19,20]; they are only “spectators” or, in other words, inactive “fillers”. Only atomically-dispersed gold cations are the active species, even if they contribute only 3%–10% to the total gold content [19]. These cations were characterized (XPS, EXAFS, XANES, TPR, ac-HAADF/STEM, DFT, *etc.*), and their catalytic activity was tested after removing weakly-bound clusters and nanoparticles (spectators) by cyanide leaching.

In previous works of our group, the activity of gold catalysts containing particles in the range of 20–700 nm in CO hydrogenation below 400 °C could be attributed to the presence of subnanometer clusters [12,13]. The existence of these clusters was revealed by combining methods, such as UV-VIS, HRTEM and calculations of XRD data with Rietveld refinement [21,22,23]. Conversely, gold nanoparticles were found to be active for the water-gas shift reaction in other works. Their activity depended on the particle size and the type of interactions established with the support [24,25,26,27]. Furthermore, the catalytic activity strongly depended on the method of preparation that, in turn, affected the support used for dispersing the metal.

In contrast to gold, works dedicated to silver subnanometer species are very few. Currently, studies by different groups explain the activity of the silver catalysts supported on SiO_2_ and Al_2_O_3_ by the presence of subnanometer silver clusters, which were detected by the FTIR of adsorbed CO (FTIR CO) [28,29,30,31,32] and UV-VIS [32,33,34].

The present work aims to further the field of catalytic properties of subnanometer species and methods to detect them. Our specific goal was to investigate the catalytic properties of silver deposited on active supports composed by TiO_2_, modified with oxides of Mg, Fe and Ce, for CO oxidation. This is an important reaction from both a fundamental viewpoint and practical application to, among others, environmental protection, safety in industrial and residential premises and inside vehicles. The differences found among the catalysts in sensitivity to redox pretreatments could not be explained considering the Ag particle size distributions measured by HRTEM in the interval 1–12 nm, but were explained by the existence of Ag species with size <1 nm non-visible in HRTEM and their interaction with supports.

## 2. Results and Discussion

**Figure 1:**
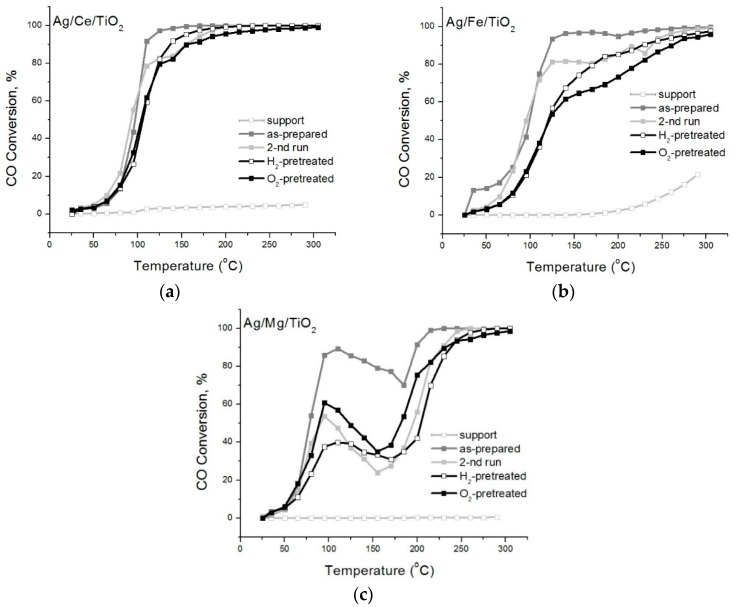
CO conversion *vs.* temperature on Ag catalysts on TiO_2_ support modified with Ce (**a**); Fe (**b**) or Mg (**c**).

Figure 1 shows the results of catalytic tests for as-prepared silver catalysts, the second run and after redox pretreatments. According to the literature, oxides, in particular ceria and titania, are not simple spectators, as they can affect the catalytic activity by participating directly in the reaction [31,32] or by modifying the chemical properties of the supported metal [35,36]. However, it can be seen in our case that bare Ce/TiO_2_ and Mg/TiO_2_ supports do not show significant conversion under the conditions studied. For Ag/Ce/TiO_2_, the light-off curves of catalytic reaction matched the typical S-shaped curve. For Ag/Mg/TiO_2_, there is a combination of a curve with a maximum (at low temperatures) and an S-shaped curve (at high temperatures). This effect, caused by both supported silver [2,16] and gold [37,38,39,40,41] catalysts, is explained in the literature as due to the activity of small clusters and larger particles, at low temperatures and at high temperatures, respectively. It can be seen that light-off curves for as-prepared Ag/Ce/TiO_2_ and Ag/Fe/TiO_2_ samples are similar; consequently, the contents of active species in them were comparable (Figure 1). Pretreatments revealed differences in their catalytic behaviors. Light-off curves for Ag/Mg/TiO_2_ samples as-prepared and after different pretreatments vary considerably, while those for Ag/Ce/TiO_2_ turn out to be almost identical. The changes among all light-off curves for Ag/Fe/TiO_2_ represented an intermediate case.

Different complementary physical and chemical characterization methods were used to investigate the causes of the differences in catalytic behavior. Table 1 shows that the specific surface area of modified supports and catalysts are similar, and the content of Ag is identical for all catalysts, which ruled out these characteristics (S_BET_ and Ag content) as the source of their distinct catalytic performance.

**Figure 2:**
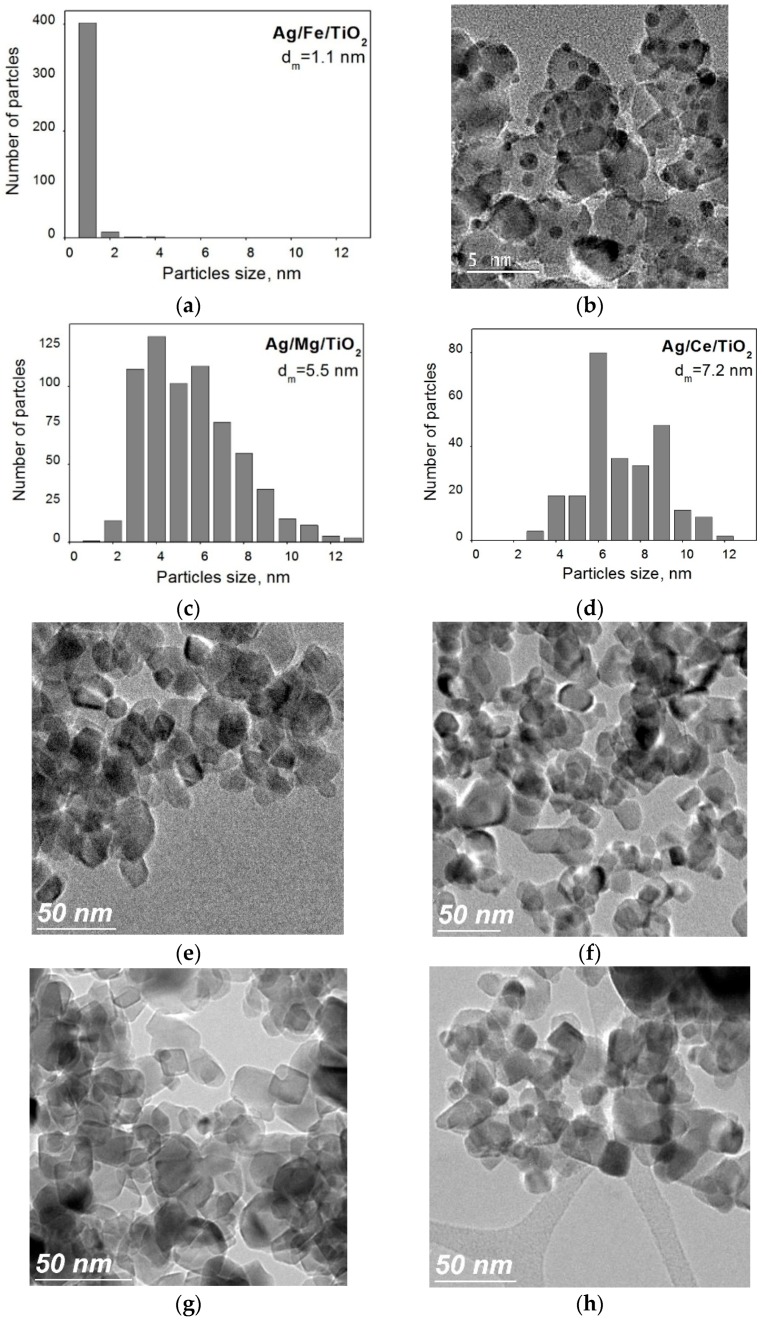
Particle size distribution of Ag catalysts supported on Fe/TiO_2_ (**a**); Mg/TiO_2_ (**c**) and Ce/TiO_2_ (**d**) treated in H_2_ at 300 °C for 1 h. Microphotographs of catalyst Ag/Fe/TiO_2_ (**b**) and supports: Fe/TiO_2_ (**e**); Mg/TiO_2_ (**f**); Ce/TiO_2_ (**g**) and TiO_2_ (**h**).

The particle size distribution for silver catalysts, calculated from HRTEM images, is displayed in Figure 2. An extremely narrow particle size distribution centered at 1 nm is observed for Ag/Fe/TiO_2_ (Figure 2a,b). The histogram for Ag/Mg/TiO_2_ (Figure 2c) is broader, with an average size of 5.5 nm. The range of particle sizes (3–12 nm) of Ag/Ce/TiO_2_ (Figure 2d) is also broad and shifted to larger sizes compared to the other samples, with an average particle size of 7.2 nm. HRTEM of the modified supports is presented in Figure 2e–g. For all studied supports, no contrasting particles related to modifiers were found on TiO_2_. Consequently, it can be inferred that modifiers are distributed homogeneously in the form of subnanometer species.

**Figure 3:**
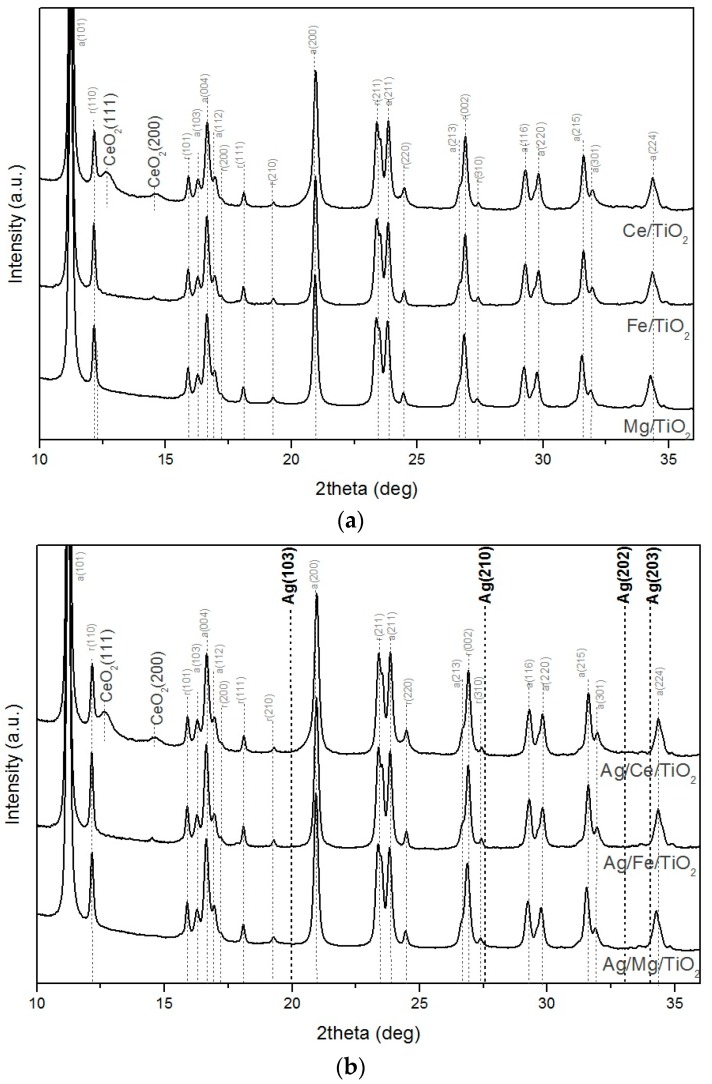
The SR-XRD patterns for the supports (**a**) and catalysts treated in H_2_ flow at 300 °C for 1 h (**b**).

Figure 3 shows the diffraction lines for the modified supports and Ag catalysts. It can be seen that SR-XRD patterns are practically the same for modified supports and the corresponding Ag catalysts. For all catalysts, reflections corresponding to the crystalline silver phase were not detected in their SR-XRD patterns (Figure 3). This suggests that silver is either amorphous or that its crystallite size is below the SR-XRD detection limit (2 nm). These results confirm the HRTEM data for the Fe-modified samples: all particles in Ag/Fe/TiO_2_ has a size of 2 nm or less.

As the particle sizes’ range recorded by the HRTEM was 3–12 nm, *i.e.*, larger than the SR-XRD detection limit, one could expect silver phases in Ag/Ce/TiO_2_ to be detectable by SR-XRD; however, the diffraction lines for silver were absent. This indicates that the silver crystallites’ size is <2 nm and that the HRTEM histogram is representative of only a small part of the silver present in Ag/Ce/TiO_2_.

**Figure 4:**
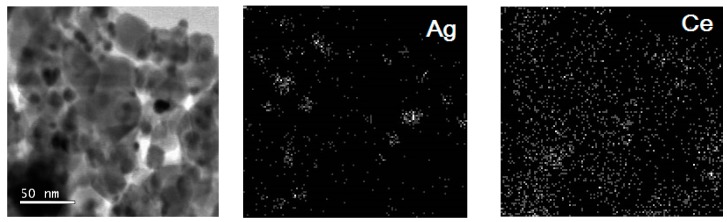
Low-magnification HRTEM micrograph and corresponding elemental maps in false colors for Ag and Ce extracted from the EDS data cube for Ag/Ce/TiO_2_ sample treated in H_2_ flow at 300 °C for 1 h.

What could cause the formation of large particles in Ag/Ce/TiO_2_ is an interesting question. From low-magnification HRTEM micrograph and corresponding elemental maps in false colors for Ag and Ce extracted from the EDS data, it can be seen that silver in Ag/Ce/TiO_2_ corresponds to both large and small particles (Figure 4). Cerium is distributed over the surface quite uniformly, which is in agreement with the HRTEM of Ce/TiO_2_. Elemental maps in false colors for Ag and Ce extracted from the EDS data and HRTEM of Ce/TiO_2_ indicate the absence of large CeO_2_ particles. It allows attributing the CeO_2_ diffraction line of very low relative intensity (Figure 3) to ~10-nm CeO_2_ crystallites, which represent only a small part of CeO_2_ total content. Hence, 3–12-nm particles of the HRTEM histogram of Ag/Ce/TiO_2_ correspond to Ag particles, not to CeO_2_ ones. Nevertheless, it should be taken into account that small silver particles were also registered by the EDS data.

Figure 3 shows that the reflections corresponding to the modifier were only detected for Ag/Ce/TiO_2_. Despite the fact that the molar concentrations of the modifiers were the same in the three modified supports, the higher molecular weight of CeO_2_ resulted in a higher mass ratio, which exceeds the weight content of other additives and explains the appearance of the corresponding peaks. CeO_2_ is in the form of large particles (~10 nm, as estimated by the Scherrer method).

To confirm the hypothesis, based on SR-XRD results, of the presence of silver species with size <1 nm in Ag/Ce/TiO_2_, we took advantage of a DRS method [41,42,43,44]. It provides an easy registration of silver clusters of <1 nm by absorption bands around 260–290 nm, Ag+ cations at 250 nm and nanoparticles at about 400 nm. Particles of 1–1.5 nm (quasi-metallic particles) are characterized by unstructured absorbance above 400 nm. The absorption characteristic of these quasi-metallic species is similar to that of colloidal silver as a consequence of increasing particle size from atomic and molecular scales to the bulk metal one [45].

**Figure 5:**
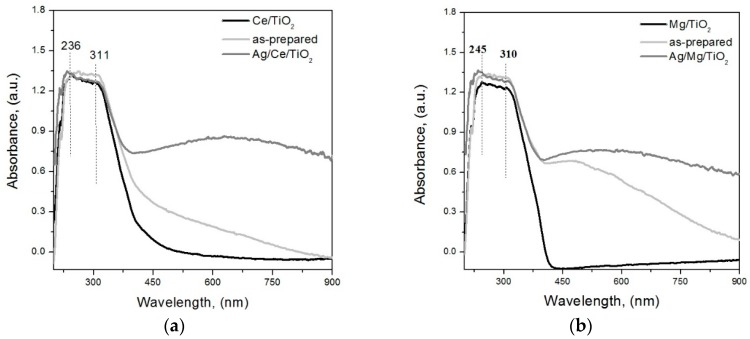

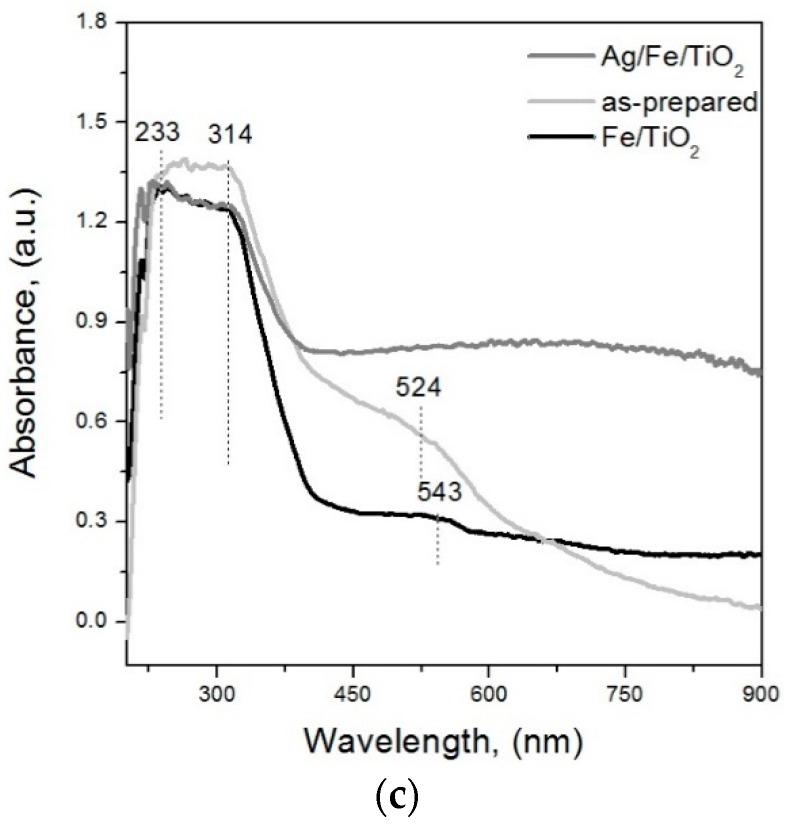
DRS of the supports based on TiO_2_, modified with Ce (**a**); Mg (**b**) or Fe (**c**) oxides and their corresponding catalysts before (as-prepared) and after reduction in H_2_ under 300 °C for 1 h.

As can be seen in Figure 5, the intense absorption peaks of the support (200–300 nm) are observed in the same region as those of Ag cations and clusters for the catalysts. This hinders the application of this method for investigating these Ag species. Catalysts after H_2_ treatment exhibit the unstructured absorbance corresponding to quasi-metallic silver particles with a size of about 1–1.5 nm. As no significant differences were observed in the visible range of the spectra of all catalysts, it can be concluded that the differences in activity after H_2_ pretreatments are not caused by quasi-metallic silver particles with a size of about 1–1.5 nm. DRS results (as SR-XRD) also indicate that in all samples, most of the Ag is in the form of species with size <1.5 nm. The large contribution of Ag species with size <1.5 nm in the three catalysts, measured by DRS, is in good agreement with the HRTEM histogram for Ag/Fe/TiO_2_ (Figure 2a). However, for Ag/Ce/TiO_2_ and Ag/Mg/TiO_2_, these DRS data show that their HRTEM histograms represent only part of the actual sizes distributions, and they do not include Ag species <1.5 nm.

**Figure 6:**
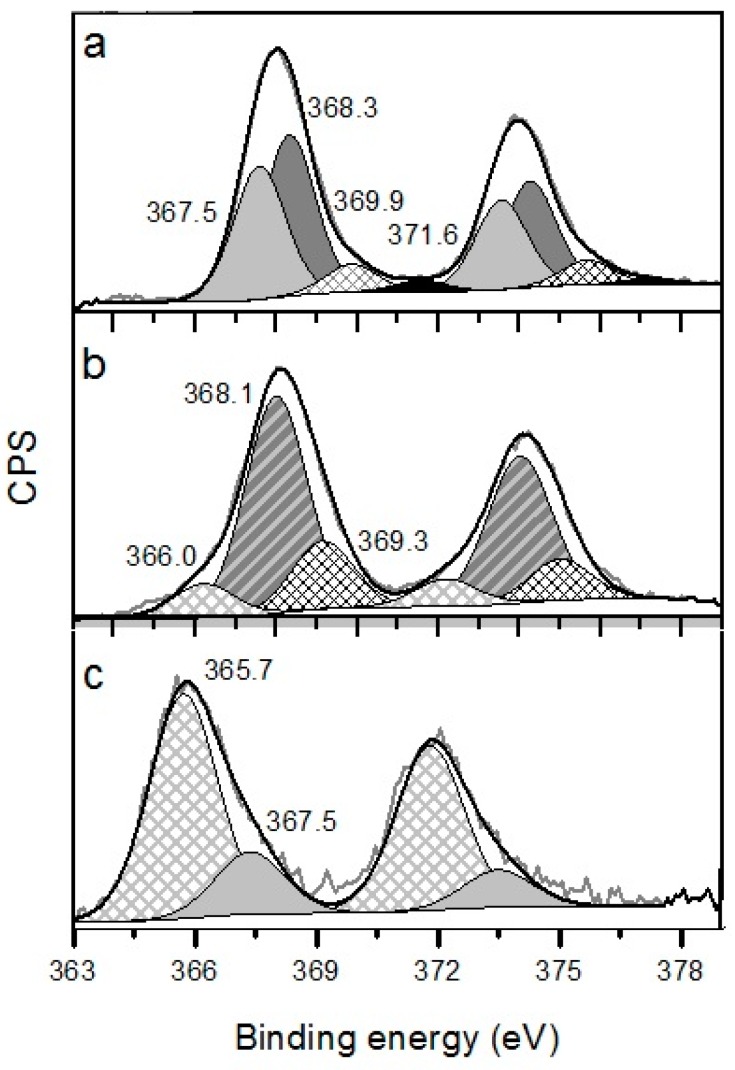
XPS of lines Ag 3d5/2 for Ag catalysts supported on Mg/TiO_2_ (**a**); Fe/TiO_2_ (**b**) and Ce/TiO_2_ (**c**) after reduction in H2 at 300 °C for 1 h.

XPS spectra of the Ag 3d5/2 lines of the catalysts are presented in Figure 6. Ag 3d5/2 peaks required deconvolution for all samples, because their widths exceed those corresponding to a single state. Table 2 shows that most silver (80%) in Ag/Ce/TiO_2_ is in cationic form (BE = 365.7 eV [46,47,48]), while in Ag/Fe/TiO_2_, its contribution is 14%, and in Ag/Mg/TiO_2_, cationic silver was not registered. Supports modified with Ce and Fe oxides are able to stabilize cationic silver, because these additives exhibit electron-acceptor properties, while Mg oxide is electron-donor [29,44,49,50]. Furthermore, note that ceria nanoparticles are able to provide oxygen dissociation, pointing out differences between Ag/Ce/TiO_2_ and Ag/Fe/TiO_2_ regarding their catalytic properties [36,51,52].

**Figure 7:**
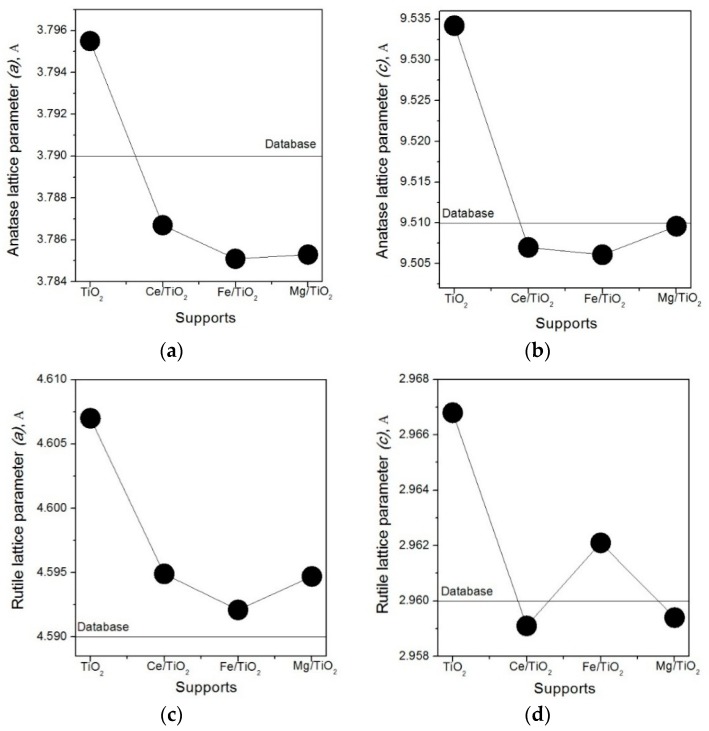
Lattice constants of anatase (**a**,**b**) and rutile (**c**,**d**) calculated on the basis of EXAFS data for the studied supports.

XPS results indicate that the extent of strong metal-support interaction (SMSI) for Ag follows the trend: Ag/Ce/TiO_2_ > Ag/Fe/TiO_2_ > Ag/Mg/TiO_2_. The nature of SMSI for Ag species is discussed in more detail below. It should be noted that the initial TiO_2_ support is a mixture of two phases: rutile and anatase. Figure 7 shows that all of the lattice constants of both phases, obtained from the EXAFS data, of the initial TiO_2_ decreased when modifiers were added. Taking into account the low modifier content (<6 wt %), one may conclude that modifiers are quite uniformly distributed throughout the TiO_2_ structure and that they strongly interact with TiO_2_. EXAFS results also showed that the rutile phase contribution increased 4%, 5% and 6% after modification with Ce, Fe and Mg oxide, respectively. The change of the rutile/anatase ratio (bulk properties) after modification is known to occur [53]. Details of the discussion of the influence of modification on the rutile/anatase ratio and lattice constants exceed the allocated extension of this paper and will be published elsewhere.

**Figure 8:**
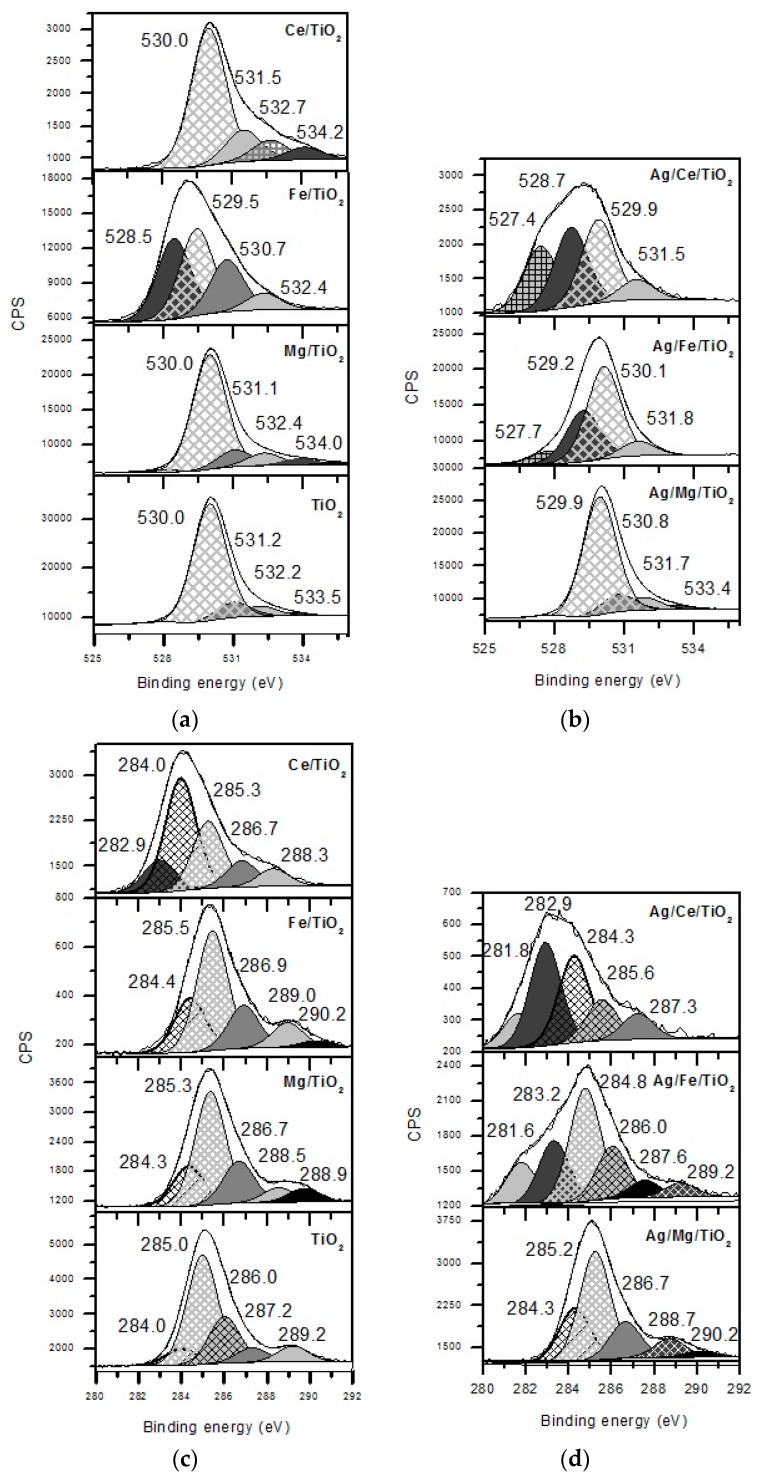
XPS lines of O1s (**a**,**b**) and C1s (**c**,**d**) for supports (**a**,**c**) and Ag catalysts after reduction in H_2_ at 300 °C for 1 h (**b**,**d**).

The XPS study of various forms of oxygen and carbon showed differences in the number and contribution of oxygen and carbon surface states on the modified supports (Figure 8a,с). While for TiO_2_, Mg/TiO_2_ and Ce/TiO_2_, very similar C1s and O1s profiles, for Ag/Fe/TiO_2_, a big change in both is observed, indicating a strong interaction of iron oxide with the original support. Hence, the interaction modifier-TiO_2_ is the strongest for Ag/Fe/TiO_2_.

Silver addition caused redistribution of oxygen and carbon states (Figure 8b,d). New peaks of C1s and O1s with a significant contribution appeared for Ag/Ce/TiO_2_ and Ag/Fe/TiO_2_, while for Ag/Mg/TiO_2_, minimal changes of the investigated surface states are observed compared to TiO_2_ and Mg/TiO_2_. This proves the SMSI in the Ag/Ce/TiO_2_ and Ag/Fe/TiO_2_ catalysts and a weaker interaction in Ag/Mg/TiO_2_. This can be the reason for the formation of small quasi-metallic silver particles with a size of 1 nm on Fe/TiO_2_ and even smaller subnanometer silver species on Ce/TiO_2_.

**Figure 9:**
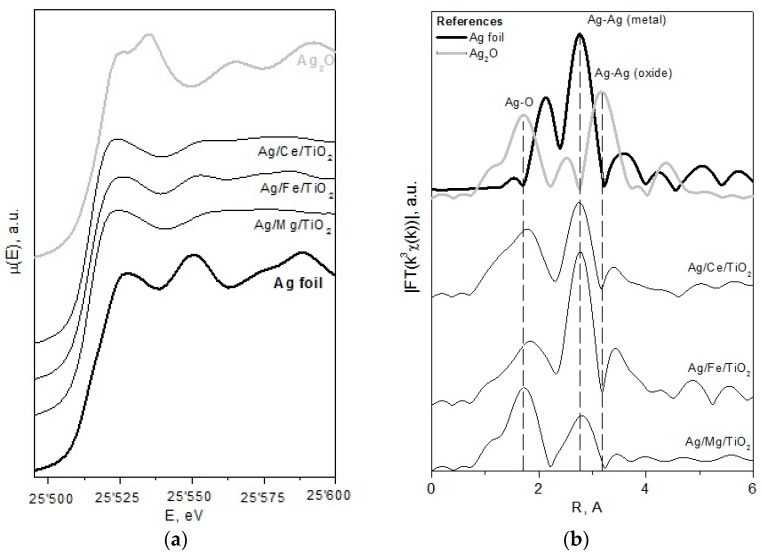
XANES (**a**) and EXAFS (**b**) spectra of catalysts treated in H_2_ flow at 300 °C for 1 h.

The results of XANES and EXAFS methods for the studied catalysts and reference compounds (metallic silver and bulk silver oxide) are presented in Figure 9, and their evaluation is in Table 3. For these methods, we focus our attention on analyzing the results for Ag/Fe/TiO_2_ and Ag/Mg/TiO_2_. Data for Ag/Ce/TiO_2_ were not analyzed, because XPS data (Table 2) showed that 80% of Ag is in the cationic form, not registered by XANES and EXAFS methods. Ag_2_O species with coordination numbers 2.5 and 2.6 were detected for Ag/Fe/TiO_2_ and Ag/Mg/TiO_2_, respectively. This indicates that Ag_2_O species are very small. Their contribution is higher in Ag/Mg/TiO_2_ (90 mol %) compared to Ag/Fe/TiO_2_ (62 mol %). Consequently, there are smaller particles in Ag/Mg/TiO_2_. The Ag/Fe/TiO_2_ sample contains more metallic particles characterized by a significantly higher coordination number (five), which is closer to that of bulk metal (12) than that for Ag/Mg/TiO_2_ (2.2).

This evidences that metallic particles in Ag/Mg/TiO_2_ are smaller than in Ag/Fe/TiO_2_, which is again in contradiction to HRTEM data (Figure 2). This in turn indicates that Ag species of less than 1 nm do exist in Ag/Mg/TiO_2_, but they were not registered by HRTEM. These species can include cations (as shown by XPS data), as well as metallic and oxide clusters (as evidenced from XPS, XANES and EXAFS data). Coordination numbers from EXAFS data indicate that the silver particle sizes in Ag/Ce/TiO_2_ and Ag/Mg/TiO_2_ are similar, whereas those in Ag/Fe/TiO_2_ are larger. This is in agreement with DRS, SR-XRD and XPS data. Hence, the analysis of the results of all applied methods showed that HRTEM histograms of Ag/Ce/TiO_2_ and Ag/Mg/TiO_2_ only reflect part of the Ag particles, those are excluding subnanometer Ag species.

Turning back to XPS data, it is worthy to note that the position of the Ag 3d5/2 peak for Ag/Fe/TiO_2_ (368.1 eV) is on the limit of the typical positions for Ag_2_O and Ag^0^. Probably, this Ag 3d5/2 peak is typical for 1 nm Ag quasi-metallic particles, which composes the main part of this sample. We did not find in the literature data for Ag 3d5/2 BE for 1-nm Ag quasi-metallic particles. Therefore, probably, our data can be considered as some of the first ones for their characterization. The interpretation of the peaks below 369 eV observed for Ag/Fe/TiO_2_ and Ag/Mg/TiO_2_ is also difficult, because this BE interval is not well identified in the literature. Kim’s group [54,55] suggested that this interval is characteristic for Ag clusters with size <2 nm supported on highly ordered pyrolytic graphite. Thus, Ag/Fe/TiO_2_ contains mainly quasi-metallic Ag particles of 1 nm, those <2 nm Ag clusters and a small contribution of silver cations. Thus, for this catalyst, HRTEM and XPS data are in good agreement. Spectra of Ag/Mg/TiO_2_ can be interpreted as composed by Ag^0^ (BE = 368.3 eV [48]), Ag_2_O (BE = 367.5 eV [48]) and clusters with a size <2 nm. XPS data showed that 80% of surface silver is in the Ag+ state. This is in good agreement with HRTEM of Ce/TiO_2_, EDS, DRS and SR-XRD.

Oxidized silver states were observed for all catalysts. This confirms the effective interaction of silver with supports. As Fe and Ce oxides exhibit electron-acceptor properties, while Mg oxide has electron-donor ones, their interaction with silver can modify its oxidation state. For Ag/Mg/TiO_2_, the transfer of electron density from Mg oxide to silver occurs with stabilization of 52% of silver in the reduced state, and the contribution from Ag+ is not detected. In contrast, in Ag/Ce/TiO_2_, 80% of surface silver is stabilized as Ag+. Ag/Fe/TiO_2_ is an intermediate case; only 14% of silver is in the form of Ag+ cations. In Ag/Ce/TiO_2_ catalyst, 100% of the surface silver is in oxidized states. These results confirm that the HRTEM Ag/Ce/TiO_2_ histogram (Figure 2d) corresponds only to a small part of silver particles.

Catalysts’ investigation by these physicochemical methods showed that, firstly, the HRTEM histogram for Ag/Fe/TiO_2_ (Figure 2) is the most exact, while for Ag/Mg/TiO_2_ and Ag/Ce/TiO_2_, besides Ag nanoparticles with sizes from 1–12 nm, Ag species with size <1 nm, non-visible in HRTEM, exist. Thus, the majority of Ag species in all samples has sizes ≤1 nm. Secondly, the strength of interaction between Ag and support decreases as follows: Ag/Ce/TiO_2_ > Ag/Fe/TiO_2_ > Ag/Mg/TiO_2_.

The majority of Ag species in all samples have size ≤1 nm; consequently, differences in the catalytic activity cannot be explained by differences in Ag species size, but can be caused by the difference in the SMSI. For Ag/Ce/TiO_2_, the activity was very stable for all pretreatments (Figure 1). This can be explained by the fact that this sample has the most SMSI. On the contrary, for Ag/Mg/TiO_2_, activity was very sensitive to the type of pretreatment. This can be due to the weak interaction of Ag species with support. The behavior of Ag/Fe/TiO_2_ presents an intermediate case as far as catalytic activity and SMSI are concerned.

The existence of small silver species, active for an industrially-important reaction, such as CO oxidation, has been suggested in other works [1,28]. In this work, Ag subnanometer species in Ag/Mg/TiO_2_ and Ag/Ce/TiO_2_ catalysts were for the first time detected and then characterized by using several physicochemical techniques. The catalytic performance can actually be enhanced if deactivation of subnanometer silver species, that are active at low temperatures, is prevented. This prevention can be done by increasing of SMSI by way of selecting the type of modifier and improving its distribution on the support surface.

This work represents the continuation of studies where catalytic properties of silver catalysts could not be explained by particle size distributions measured by HRTEM and are explained by existence of small silver species. Works dedicated to this matter are very few; we found only four publications [12,13,14,15]. Below, we briefly describe them.

Undetected small silver clusters have been suggested as the source of specific catalytic properties [1,2,3,4]. Flytzani-Stephanopoulos *et al.* [33] explained the catalytic properties in the reduction of NO with methane by the coexistence of highly dispersed clusters (<2 nm) of oxidized silver and embedded Ag ions, which are active in selective reduction of CH_4_, but low active in CH_4_ combustion. The presence of small clusters was detected by the absorption band at 350 nm, typical for Ag_n_^δ+^ in diffuse reflectance UV spectra. The unusual behavior of silver on SiO_2_ catalysts was observed after oxidizing and reducing pretreatments in CO oxidation [28]. During reduction in H_2_ at 100–300 °C, oxidized silver species were redispersed, leading to the remarkable activity in CO oxidation. The existence of small silver species was detected by the FTIR of adsorbed CO [28]. It was shown that silver species could not be completely reduced to the metallic state, even after reduction by H_2_ at 500 °C.

The present work shows deep insights into the existence of Ag species with size ≤1 nm, non-detectable in HRTEM, as in the works previously mentioned. Combination of HRTEM, SR-XRD, DRS, XPS, EXAFS and XANES methods allowed us to detect and characterize these Ag species and to estimate the strength of their interaction with supports. These results led to concluding that the sensitivity of CO oxidation catalysts to pretreatments depends on the strength of the interaction of Ag species with supports. The combination of these methods can be used not only for detection and characterization of subnanometer metallic species in catalysts, but also for other materials used in various fields, such as sensors [56].

Activation-deactivation of the active component after different pretreatments can be caused by many factors, such as encapsulation, agglomeration, redispersion, carbonization, reduction-oxidation, *etc.* In the case of small particles, in addition to these factors, deactivation due to SMSI that is not strong enough should not be forgotten.

Under conditions that favor the formation of small supported species (say, high specific surface areas of supports, low metal concentration and active supports), the particle size distribution obtained from HRTEM results can be apparent, in other words, not correct. They can represent only part (in some cases, an extremely low part [29]) of all of the supported metal, because HRTEM registers particles ≥1 nm and does not register those ones with size <1 nm. Analysis and discussions of catalytic data taking into account these histograms can lead to the wrong conclusions. With the more widespread application of ac-HAADF/STEM to identify subnanometer species, it is expected that soon, this technique will become a routine procedure. At the moment, only a few researchers use this method for catalyst characterization. However, even in the case of the application ac-HAADF/STEM, its combined use with XPS, DRS and EXAFS is still much recommended, because they allow measuring a relatively big amount of the material and provide average characteristics, while ac-HAADF/STEM can be non-representative, especially for samples with an inhomogeneous component distribution. Moreover, complimentary information about charge, oxidation state, *etc.*, obtained by these techniques would be enriching.

This work is one of the few paying attention to the problem of the identification and characterization of subnanometer species undetectable by HRTEM. This phenomenon should take the adequate place in nanoscience.

## 3. Materials and Methods

### 3.1. Catalysts Preparation

Titania Degussa P25 (Evonik’s Chemicals Business Area, Essen, Germany) was used as the support (45 m^2^·g^−1^, nonporous, 70% anatase and 30% rutile, purity >99.5%). Before preparation, titania was dried in air at 100 °C for at least 24 h. Modification of titania with a molar ratio Ti/modifier = 40 was made by impregnation (2.5 cm^3^/g) of initial TiO_2_ with aqueous solutions of modifier (M) precursors, Ce(NO_3_)_3_·6H_2_O, Fe(NO_3_)_3_·9H_2_O and Mg(NO_3_)_2_, from Aldrich (Aldrich, St. Louis, MO, USA). Impregnated supports were dried at room temperature for 48 h and then at 110 °C for 4 h and finally calcined at 550 °C for 4 h.

Commercial AgNO_3_ from Aldrich was used as the silver precursor. Catalysts Ag/M/TiO_2_ with 2.2 wt % Ag nominal loading were prepared by deposition precipitation with NaOH in the absence of light following the previously-reported procedure [57,58,59].

### 3.2. Catalytic Tests

For catalytic CO oxidation measurements, 0.5 g of the catalyst were packed in a quartz flow reactor. A first run of reaction was accomplished with as-prepared samples. Then, catalysts were cooled down in a reaction mixture, and then, the second run was performed. Alternatively, pretreatments in hydrogen or oxygen flow (30 mL/min) at 300 °C for 1 h were applied to the catalyst prior to the test. The catalytic reaction was conducted with a 200-mL/min flow rate of the reactant gas mixture 1% vol. O_2_, 1% vol. CO in Ar and with temperature increase from 30–305 °C in 15 °C steps every 20 min. The products were analyzed by gas chromatograph CHROMOS GC–1000, equipped with a TCD, and using two separate packed columns filled with CaA (to analyze oxygen and hydrogen) and AG-3 sorbent (to analyze both carbon oxides), respectively, and He as the carrier gas.

### 3.3. Samples’ Characterization

Catalysts, either as-prepared or pretreated in hydrogen at 300 °C for 1 h, were studied by diffuse reflectance UV-VIS spectroscopy (DRS) with a CARY 300 SCAN (Varian, Palo Alto, CA, USA) spectrophotometer. Optical spectra presented in this work were obtained by subtraction of the spectra of pure supports from the spectra of silver samples.

Prior to any characterization described below, the samples were pretreated in hydrogen at 300 °C for 1 h. The textural properties of samples were determined from nitrogen adsorption-desorption isotherms (−196 °C) recorded with a TriStar 3000 apparatus (Micromeritics, Norgross, GA, USA). Prior to experiments, samples were degassed at 300 °C in a vacuum for 5 h. 

Silver contents were measured by energy dispersive spectroscopy (EDS) in the JEOL-5300 scanning electronic microscope with a Kevex Superdry detector (JEOL, Tokyo, Japan). High resolution transmission electron microscopy (HRTEM) studies were carried out using a JEM 2100F microscope operating with a 200-kV accelerating voltage. The samples were ground into a fine powder and dispersed ultrasonically in hexane at room temperature. Then, a drop of the suspension was put on a lacey carbon-coated Cu grid. At least ten representative images were taken for each sample. A particle size distribution was obtained by counting *ca*. 100 particles for each sample. Сhemical analysis of surface carried out by spectroscopy of dispersion energy (EDS) with X-Max detector (80 mm^2^) (Oxford Instruments, Oxfordshire, UK).

Synchrotron radiation X-ray diffraction (SR-XRD) experiments were carried out as described in [60]. Diffraction patterns of powdered materials were taken in transmission at λ = 0.68886 Å, using an Imaging Plate 2D detector (exposure time: 30 min). In some cases, full Rietveld analysis [61,62] was carried out using the Jana2006 program [60].

XAFS experiments were carried out as described in [60]. The XANES spectra were taken in transmission. Primary processing of XAFS spectra was done using the IFEFFIT software package [63,64]. Extended analysis (EXAFS) was carried out only for the EXAFS spectra taken *in situ* at room temperature. The Fourier transforms were analyzed for *k* = 2.0–11.6 Å^−1^ with the weight coefficient *k*^3^ using the phases and amplitudes of photoelectrons scattering calculated in terms of the FEFF8 software [65].

The samples were characterized by X-ray photoelectron spectroscopy (XPS) with a SPECS GmbH custom-made system using a PHOIBOS 150 WAL hemispherical analyzer and a non-monochromated X-ray source (*SPECS* Surface Nano Analysis GmbH, Berlin, Germany). All of the data were acquired using Al Kα X-rays (1486.6 eV, 200 W). A pass-energy of 50 eV, a step size of 0.1 eV/step and a high-intensity lens mode were selected. The diameter of the analyzed area was 3 mm. Charging shifts were referenced against the Ti 2p3/2 peak of TiO_2_ at 458.8 eV. The pressure in the analysis chamber was maintained lower than 1 × 10^−8^ mbar. The accuracy of the binding energy (BE) values is ±0.1 eV. Spectra are presented without smoothing or background subtraction, with intensity in counts per second (CPS). Peak areas were estimated by calculating the integral of each peak after subtracting a Shirley-type background and fitting the experimental peak to a combination of Lorentzian/Gaussian lines with a 30/70 proportion and keeping the same width on all lines.

## 4. Conclusions

Differences in sensitivity to oxidative and reductive pretreatments was revealed for Ag/Fe/TiO_2_, Ag/Mg/TiO_2_ and Ag/Ce/TiO_2_ catalysts in CO oxidation. The observed phenomena could not be explained taking into account the Ag particle size distributions measured by HRTEM in the interval 1-13 nm. However, they can be explained by the existence of Ag species with size <1 nm, non-visible in HRTEM, and their interaction with the supports. The present work is one of the few to address the problem of the identification and characterization of such subnanometer species undetectable by HRTEM and highlights their role as active species and the need to take them into account to understand integrally the catalysis by supported nanometals.

## Figures and Tables

**Table 1:** Textural properties of supports and catalysts and analytical silver content of the catalysts treated in H_2_ at 300 °C for 1 h.

Samples	S_BET_, m^2^/g		EDX
	Support	Catalyst	Ag Content, wt %
Ag/Ce/TiO_2_	43.4	46.3	2.1 ± 0.4
Ag/Fe/TiO_2_	45.5	44.0	1.9 ± 0.4
Ag/Mg/TiO_2_	42.6	43.9	2.0 ± 0.3

**Table 2:** Binding energy and relative atomic concentrations of various silver electronic states in catalysts.

Catalysts	BE of Different gold Electronic States (eV) and Their Relative Atomic Concretions, % (Indicated in Parenthesis)			
	Ag^+^	Ag_2_O	Ag°	Ag <2 nm
	364.0–366.2	367.4–368.1	368.1–368.4	>369.0
Ag/Mg/TiO_2_	-	367.5 (47%)	368.3 (40%)	369.9 (9%), 371.6 (4%)
Ag/Fe/TiO_2_	366.0 (14%)	368.1 (65%)		369.3 (21%)
Ag/Ce/TiO_2_	365.7 (80%)	367.5 (20%)	-	-

**Table 3:** Sample composition and structural parameters obtained from XAFS spectroscopies data.

Sample	XANES			EXAFS		
	Phase	Molar Fraction, %	R-Factor, %	Corresponding Single-Scattering Path	Coordination Number	Mean Squared Displacement
Ag foil	Ag	100	-	Ag-Ag	12	0.0097
Ag_2_O	Ag_2_O	100	-	Ag-O	4	-
Ag/Ce/TiO_2_	Ag	26.4	0.030	Ag-Ag	2.7	0.0115
	Ag_2_O	73.6		Ag-O	3.5	0.0183
Ag/Mg/TiO_2_	Ag	9.8	0.024	Ag-Ag	2.2	0.0149
	Ag_2_O	90.2		Ag-O	2.6	0.0127
Ag/Fe/TiO_2_	Ag	38.3	0.025	Ag-Ag	5.0	0.0131
	Ag_2_O	61.7		Ag-O	2.5	0.0170

## Abbreviations

The following abbreviations are used in this manuscript:

ac-HAADF/STEM	aberration-corrected high angle annular dark field scanning transmission electron microscopy
BE	binding energy
CPS	Counts per second.
DFT	density functional theory
DRS	diffuse reflectance spectroscopy in ultraviolet and visible diapason
EDS	energy dispersive spectroscopy
EXAFS	extended X-ray absorption fine structure
FTIR CO	Fourier-transform infrared spectroscopy of adsorbed CO.
HRTEM	high resolution transmission electron microscopy
SMSI	strong metal-support interaction
SR-XRD	synchrotron radiation X-ray diffraction
TCD	thermal conductivity detector
TPR	temperature-programmed reduction
UV-VIS	ultraviolet-visible spectroscopy
XPS	X-ray photoelectron spectroscopy,
XANES	X-ray absorption near edge structure
WGS	water-gas shift reaction

## References

[1] Zhu H., Ma Z., Clark J.C., Pan Z., Overbury S.H., Dai S. (2007). Low-temperature CO oxidation on Au/fumed SiO_2_-based catalysts prepared from Au(en)_2_Cl_3_ precursor. Appl. Catal. A..

[2] Bondarchuk I.S., Mamontov G.V. (2015). Role of PdAg Interface in Pd–Ag/SiO_2_ Bimetallic Catalysts in Low Temperature Oxidation of Carbon Monoxide. Kinet. Catal..

[3] Mamontov G.V., Knyazev A.S., Paukshtis E.A., Vodyankina O.V. (2013). Adsorption and conversion of ethylene glycol on the surface of Ag containing catalyst modified with phosphate. Kinet. Catal..

[4] Martynova D.O., Kibis L.S., Stonkus O.A., Vodyankina O.V., Izaak T.I., Slavinskaya E.M., Boronin A.I. (2013). Synthesis and Catalytic Activity of Porous Blocked Ag/SiO_2_ Composites in Low Temperature Carbon Monoxide Oxidation. Kinet. Catal..

[5] Morgan K., Inceesungvorn B., Goguet A., Hardacre C., Meunier F.C., Shekhtman S.O. (2012). TAP studies on 2% Ag/c–Al_2_O_3_ catalyst for selective reduction of oxygen in a H_2_-rich ethylene feed. Catal. Sci. Technol..

[6] Guerba H., Djellouli B., Petit C., Pitchon V. (2014). CO oxidation catalyzed by Ag/SBA-15 catalysts: Influence of the hydrothermal treatment. C. R. Chim..

[7] Inceesungvorn B., López-Castro J., Calvino J.J., Bernal S., Meunier F.C., Hardacre C., Griffin K., Delgado J.J. (2011). Nano-structural investigation of Ag/Al_2_O_3_ catalyst for selective removal of O_2_ with excess H_2_ in the presence of C_2_H_4_. Appl. Catal. A Gene..

[8] Lippits M.J., Gluhoi A.C., Nieuwenhuys B.E. (2007). A comparative study of the effect of addition of CeOx and Li_2_O on c-Al_2_O_3_ supported copper, silver and gold catalysts in the preferential oxidation of CO. Top. Catal..

[9] Imaoka T., Kitazawa H., Chun W.-J., Yamamoto K. (2015). Finding the Most Catalytically Active Platinum Clusters With Low Atomicity. Angew. Chem. Int. Ed..

[10] Berr M.J., Schweinberger F.F., Döblinger M., Sanwald K.E., Wolff C., Breimeier J., Crampton A.S., Ridge C.J., Tschurl M., Heiz U. (2012). Size-selected Subnanometer Cluster Catalysts on Semiconductor Nanocrystal Films for Atomic Scale Insight into Photocatalysis. Nano Lett..

[11] Vilar-Vidal N., Rivas J., Lopez-Quintela M.A. (2012). Size dependent catalytic activity of reusable subnanometer copper(0) clusters. ACS Catal..

[12] Pestryakov A.N., Lunin V.V., Kharlanov A.N., Bogdanchikova N.E., Tuzovskaya I.V. (2003). Electronic state of gold in supported clusters. Eur. Phys. J. D.

[13] Bogdanchikova N., Pestryakov A., Farias M.H., Diaz J.A., Avalos M., Navarrete J. (2008). Formation of TEM- and XRD-undetectable gold clusters accompanying big gold particles on TiO_2_-SiO_2_ supports. Solid State Sci..

[14] Flytzani-Stephanopoulos M., Gates B.C. (2012). Atomically Dispersed Supported Metal Catalysts. Annu. Rev. Chem. Biomol. Eng..

[15] Sun S., Zhang G., Gauquelin N., Chen N., Zhou J., Yang S., Chen W., Meng X., Geng D., Banis M.N. (2013). Single-atom Catalysis Using Pt/Graphene Achieved through Atomic Layer Deposition. Sci. Rep..

[16] Allard L.F., Flytzani-Stephanopoulos M., Overbury S.H. (2010). Behavior of Au Species in Au/Fe_2_O_3_ Catalysts Characterized by Novel *In Situ* Heating Techniques and Aberration-Corrected STEM Imaging. Microsc. Microanal..

[17] Lessard J.D., Valsamakis I., Flytzani-Stephanopoulos M. (2012). Novel Au/La_2_O_3_ and Au/La_2_O_2_SO_4_ catalysts for the water–gas shift reaction prepared via an anion adsorption method. Chem. Commun..

[18] Lee Y., He G., Akey A.J., Si R., Flytzani-Stephanopoulos M., Herman I.P. (2011). Raman Analysis of Mode Softening in Nanoparticle CeO_2-δ_ and Au-CeO_2-δ_ during CO Oxidation. J. Am. Chem. Soc..

[19] Flytzani-Stephanopoulos M. (2014). Gold Atoms Stabilized on Various Supports Catalyze the Water Gas Shift Reaction. Acc. Chem. Res..

[20] Yang M., Li S., Wang Y., Herron J.A., Xu Y., Allard L.F., Lee S., Huang J., Mavrikakis M., Flytzani-Stephanopoulos M. (2014). Catalytically active Au-O(OH)_x_ species stabilized by alkali ions on zeolites and mesoporous oxides. Science.

[21] Carabineiro S.A.C., Chen X., Martynyuk O., Bogdanchikova N., Avalos-Borja M., Pestryakov A., Tavares P.B., Orfao J.J.M., Pereira M.F.R., Figueiredo J.L. (2015). Gold supported on metal oxides for volatile organic compounds total oxidation. Catal. Today.

[22] Carabineiro S.A.C., Bogdanchikova N., Pestryakov A., Tavares P.B., Fernandes L., Figueiredo J.L. (2011). Gold nanoparticles supported on magnesium oxide for CO oxidation. Nanoscale Res. Lett..

[23] Carabineiro S.A.C., Bogdanchikova N., Avalos-Borja M., Pestryakov A., Tavares P.B., Figueiredo J.L. (2011). Gold supported on metal oxides for carbon monoxide oxidation. Nano Res..

[24] Shekhar M., Wang J., Lee W.-S., Williams W.D., Min Kim S., Stach E.A., Miller J.T., Delgass W.N., Ribeiro F.H. (2012). Size and Support Effects for the Water-Gas Shift Catalysis over Gold Nanoparticles Supported on Model Al_2_O_3_ and TiO_2_. J. Am. Chem. Soc..

[25] Rodriguez J.A., Senanayake S.D., Stacchiola D., Liu P. (2014). Hrbek, The Activation of gold and the water-gas shift reaction: Insights from Studies with model catalysts. J. Acc. Chem. Res..

[26] Hussain A., Gracia J., Nieuwenhuys B.E., Niemantsverdriet J.W. (2013). (Hans) Explicit roles of Au and TiO_2_ in a bifunctional Au/TiO_2_ catalyst for the water-gas shift reaction: A DFT Study. ChemCatChem.

[27] Boccuzzi F., Chiorino A., Manzoli M., Andreeva D., Tabakova T. (1999). FTIR Study of the low-temperature water-gas shift reaction on Au/Fe_2_O_3_ and Au/TiO_2_ Catalysts. J. Catal..

[28] Qu Z., Huang W., Cheng M., Bao X. (2005). Restructuring and Redispersion of Silver on SiO_2_ under Oxidizing/Reducing Atmospheres and Its Activity toward CO Oxidation. J. Phys. Chem. B.

[29] Pestryakov A.N., Lunin V.V. (2000). Physicochemical study of active sites of metal catalysts for alcohol partial oxidation. J. Mol. Catal. A Chem..

[30] Tabakova T., Boccuzzi F., Manzoli M., Chiorino A., Andreeva D. (2005). Characterization of nanosized gold, silver and copper catalysts supported on ceria. Stud. Surf. Sci. Catal..

[31] Boccuzzi F., Chiorino A., Manzoli M., Andreeva D., Tabakova T., Ilieva L., Iadakiev V. (2002). Gold, silver and copper catalysts supported on TiO_2_ for pure hydrogen production. Catal. Today.

[32] Keshavaraja A., She X., Flytzani-Stephanopoulos M. (2000). Selective catalytic reduction of NO with methane over Ag-alumina catalysts. Appl. Catal. B Environ..

[33] Kolobova E., Pestryakov A., Shemeryankina A., Kotolevich Y., Martynyuk O., Tiznado Vazquez H.J., Bogdanchikova N. (2014). Formation of silver active states in Ag/ZSM-5 catalysts for CO oxidation. Fuel.

[34] Bogdanchikova N., Tuzovskaya T., Pestryakov A., Susarrey-Arce A. (2011). Comparative study of formation and stabilization of gold and silver clusters and nanoparticles in mordenites. J. Nanosci. Nanotechnol..

[35] Green I.X., Tang W., Neurock M., Yates Jr J.T. (2011). Spectroscopic Observation of Dual Catalytic Sites During Oxidation of CO on a Au/TiO_2_ Catalyst. Science.

[36] Parka J.B., Graciani J., Evans J., Stacchiola D., Ma S., Liu P., Nambu A., Fernández Sanz J., Hrbeka J., Rodriguez J.A. (2009). High catalytic activity of Au/CeOx/TiO_2_(110) controlled by the nature of the mixed-metal oxide at the nanometer level. PNAS.

[37] Ma Z., Yin H., Dai S. (2010). Influence of Preparation Methods on the performance of metal phosphate-supported gold catalysts in CO Oxidation. Catal. Lett..

[38] Ma Z., Yin H., Overbury S.H., Dai S. (2008). Metal Phosphates as a New Class of Supports for Gold Nanocatalysts. Catal. Lett..

[39] Pestryakov A.N., Bogdanchikova N., Simakov A., Tuzovskaya I., Jentoft F., Farias M., Díaz A. (2007). Catalytically active gold clusters and nanoparticles for CO oxidation. Surf. Sci..

[40] Pestryakov A., Tuzovskaya I., Smolentseva E., Bogdanchikova N., Jentoft F.C., Knop-Gericke A. (2005). Formation of gold nanoparticles in zeolites. Int. J. Modern Phys. B.

[41] Smolentseva E., Bogdanchikova N., Simakov A., Pestryakov A., Avalos M., Farias M.H., Tompos A., Gurin V. (2007). Catalytic activity of gold nanoparticles incorporated into modified zeolites. J. Nanosci. Nanotechnol..

[42] Bogdanchikova N., Meunier F.C., Avalos-Borja M., Breen J.P., Pestryakov A. (2002). On the nature of the silver phases of Ag/Al_2_O_3_ catalysts for reactions involving nitric oxide. Appl. Catal. B.

[43] Pestryakov A.N., Lunin V.V., Bogdanchikova N.E., Petranovskii V.P., Knop-Gericke A. (2003). Supported foam-silver catalysts for alcohol partial oxidation. Catal. Commun..

[44] Pestryakov A.N., Bogdanchikova N.E., Knop-Gericke A. (2004). Alcohol selective oxidation over modified foam-silver catalysts. Catal. Today..

[45] Ershov B., Abkhalimov E., Sukhov N. (2005). Formation of Long-Lived Clusters and Silver Nucleation in the g-Irradiation of Aqueous Silver Perchlorate Solutions Containing Polyphosphate. High Energ. Chem..

[46] Rtimi S., Baghrichea O., Sanjines R., Pulgarina C., Bensimonc M., Kiwi J. (2013). TiON and TiON-Ag sputtered surfaces leading to bacterial inactivation under indoor actinic light. J. Photochem. Photobiol. A.

[47] Mejia I.M., Marín M., Sanjines R., Pulgarín C., Mielczarski E., Mielczarski J., Kiwi L. (2010). Magnetron-sputtered Ag-modified cotton textiles active in the inactivation of airborne bacteria. ACS Appl. Mater. Interfaces.

[48] Wagner C.D., Riggs M., Davis E., Müllenberg G. (1979). Handbook of X-ray Photoelectron Spectroscopy.

[49] Pestryakov A.N., Davydov A.A. (1994). Active electronic states of silver catalysts for methanol selective oxidation. Appl. Catal. A.

[50] Pestryakov A. (1996). Modification of silver catalysts for oxidation of methanol to formaldehyde. Catal. Today.

[51] Yang F., Graciani J., Evans J., Liu P., Hrbek J., Sanz J.F., Rodriguez J.A. (2011). CO Oxidation on inverse CeOx/Cu(111) catalysts: High catalytic activity and ceria-promoted dissociation of O_2_. J. Am. Chem. Soc..

[52] Rao K.N., Bharali P., Thrimurthulu G., Reddy B.M. (2011). Supported copper–ceria catalysts for low temperature CO oxidation. Catal. Commun..

[53] Kuzmicheva G.M. (2015). Nanosized phases with titanium(iv) oxides. Preparation. Characterisation. Properties. Fine Chem. Technol..

[54] Lopez-Salido I., Lim D.C., Kim Y.D. (2005). Ag nanoparticles on highly ordered pyrolytic graphite (HOPG) surfaces studied using STM and XPS. Surf. Sci..

[55] Lim D.C., Lopez-Salido I., Kim T.D. (2005). Size selectivity for CO-oxidation of Ag nanoparticles on highly ordered pyrolytic graphite (HOPG). Surf. Sci..

[56] Ahmad M.Z., Golovko V.B., Adnan R.H., Abu Bakar F., Ruzicka J., Anderson D.P., Andersson G.G., Wlodarski W. (2013). Hydrogen sensing using gold nanoclusters supported on tungsten trioxide thin film. Int. J. Hydrog. Energy.

[57] Zanella R., Giorgio S., Henry C.R., Louis C. (2002). Alternative Methods for the Preparation of Gold Nanoparticles Supported on TiO_2_. Phys. Chem. B.

[58] Zanella R., Delannoy L., Louis C. (2005). Mechanism of deposition of gold precursors onto TiO_2_ during the preparation by cation adsorption and deposition-precipitation with NaOH and urea. Appl. Catal. A.

[59] Zanella R., Louis C. (2005). Influence of the conditions of thermal treatments and of storage on the size of the gold particles in Au/TiO_2_ samples. Catal. Today.

[60] Chernyshov A.A., Veligzhanin A.A., Zubavichus Y.V. (2009). Structural materials science end-station at the Kurchatov synchrotron radiation source: Recent instrumentation upgrades and experimental results. Nucl. Instrum. Methods Phys. Res. A.

[61] Petricek V., Dusek M., Palatinus L. (2006). Jana2006: The CRYSTALLOGRAPHIC computing System. http://jana.fzu.cz/.

[62] Rietveld H.M. (1969). A profile refinement method for nuclear and magnetic structures. J. Appl. Crystallogr..

[63] Athena R.B., Hephaestus A. (2005). Data analysis for X-ray absorption spectroscopy using IFEFFIT. J. Synchrotron Rad..

[64] Newville M. (2001). IFEFFIT: Interactive XAFS analysis and IFEF fitting. J. Synchrotron Rad..

[65] Ankudinov A.L., Ravel B., Rehr J.J., Conradson S.D. (1998). Real-space multiple-scattering calculation and interpretation of X-ray-absorption near-edge structure. Phys. Rev. B.

